# Social gaze in preterm infants may act as an early indicator of atypical lateralization

**DOI:** 10.1111/cdev.13734

**Published:** 2022-02-03

**Authors:** Rachael Davis, Georgina Donati, Kier Finnegan, James P. Boardman, Bethan Dean, Sue Fletcher‐Watson, Gillian S. Forrester

**Affiliations:** ^1^ Salvesen Mindroom Research Centre University of Edinburgh Edinburgh UK; ^2^ Department of Psychological Sciences Birkbeck, University of London London UK; ^3^ Department of Immunobiology UCL Great Ormond Street Institute of Child Health London UK; ^4^ MRC Centre for Reproductive Health University of Edinburgh Edinburgh UK; ^5^ Centre for Clinical Brain Sciences University of Edinburgh Edinburgh UK

## Abstract

Visual field biases have been identified as markers of atypical lateralization in children with developmental conditions, but this is the first investigation to consider early lateralized gaze behaviors for social stimuli in preterm infants. Eye‐tracking methods with 51 preterm (33 male, 92.1% White) and 61 term‐born (31 male, 90.1% White) infants aged 8–10 months from Edinburgh, UK, captured the development of visual field biases, comparing gaze behavior to social and non‐social stimuli on the left versus right of the screen. Preterm infants showed a significantly reduced interest to social stimuli on the left versus right compared to term children (*d* = .58). Preterm children exhibit early differential orienting preferences that may be an early indicator of atypical lateralized function.

Preterm birth affects 10.6% of births worldwide (Chawanpaiboon et al., 2019), and while survival rates for preterm infants have increased significantly over the last two decades (World Health Organization, 2018), the prevalence of developmental disabilities have not decreased. Preterm birth is a leading cause of neurodevelopmental and cognitive impairments in childhood. From meta‐analysis, children and adolescents who were born at <32 weeks of gestation are estimated to have 0.89 SD lower IQ (corresponding to a difference of 12.9 IQ points) compared to children born at term (Twilhaar et al., 2018), and this magnitude of difference persists into adulthood (Eves et al., 2021). Preterm birth and very low birth weight is consistently associated with increased prevalence of diagnoses compared with the general population: autism spectrum disorder is estimated to be 4–12 times more common, and attention deficit hyperactivity disorder is estimated to affect 7%–23% of adolescents (Johnson & Wolke, 2013). Several authors have pointed out that diagnoses, low IQ, and subclinical traits established in childhood often persist across the life course (Linsell et al., 2019).

Given the effects of prematurity on development, it is crucial to identify the earliest emerging divergences from typical development that could be precursors of adverse cognitive outcomes. Therefore, understanding how prematurity affects brain development, and the early stratification of infants offers a window of opportunity for the study of interventions designed to improve outcomes.

## Lateralization differences in preterm birth

Converging data suggest that preterm birth can result in both neuroanatomic variation (Blesa et al., 2016; Galdi et al., 2020), disordered neural connectivity (e.g., Papini et al., 2016) and structural differences relating to altered cerebral lateralization (Kwon et al., 2015; Lee et al., 2021). Cerebral lateralization refers to the development of specialized processes for the left and right hemispheres of the cerebral cortex (Rogers et al., 2013). Research has demonstrated that motor‐sensory and cognitive dominances are a fundamental principle of the two hemispheres of the vertebrate brain (Rogers et al., 2013) that is preserved in modern humans and is an indication of typical brain development (Toga & Thompson, 2003). Due to the way in which the nerve fibers are contralaterally connected, one side of the brain controls the motor‐sensory processing of the opposite side of the body (Hellige, 1996). However, that is not to be confused with motor‐sensory and cognitive biases that result from cerebral lateralization (e.g., Rogers et al., 2013).

Early cortical folding and structural brain asymmetries are associated with early formation and development of typical hemispheric lateralization (Thompson et al., 2009). Studies taking place during the third trimester of gestation have demonstrated that children born at term develop the structural underpinnings of anterior and motor language regions during this time (Dubois et al., 2009). Conversely, preterm infants exhibit atypical asymmetry early in development (Thompson et al., 2009; Lee et al., 2021) including differences in inter‐hemispheric connectivity (Kwon et al., 2015) and callosal thinning of the left hemisphere, known to be crucial to language function (Rushe et al., 2004).

Research consistently reports a left visual field (LVF) bias in children and adults when processing faces or socioemotional stimuli (Yovel et al., 2008), and it is linked to right hemispheric dominance (Le Grand et al., 2003). Preterm infants can also exhibit atypical social development (Dean et al., 2021) which raises the hypothesis, could divergences in lateralization play a part in explaining this? There is currently little published developmental research investigating functional right‐hemisphere lateralization divergences, or the subsequent effect on socioemotional processing abilities in preterm infants. One opportunity to address this gap in the literature is to consider behavioral markers of brain lateralization and the relation to higher cognitive functions (Forrester & Todd, 2018), and one frequently used measure of lateralized processing and function is visual field biases.

## Left visual field biases

Findings show a bias to the LVF in face processing (e.g., Brady et al., 2005) and emotion recognition and categorization (Guo et al., 2012). There is also some neuro‐structural evidence that the social stimuli bias is present from birth (e.g., Buiatti et al., 2019)—which may not be surprising if we believe that our social biases have been exapted from early vertebrate predator behaviors. This is also consistent with a left hemisphere bias for motor behavior (e.g., in utero, thumb sucking is highly correlated with later hand dominance for fine motor actions)—where behavior is taken as a proxy for brain organization.

The above research also suggests that a LVF bias is a behavioral marker of hemispheric lateralization for emotional processing (De Renzi, 1994). Information from the LVF is contralaterally projected to the right hemisphere, which is known to be optimized for social and emotional processing (Vallortigara et al., 2011). This bias has since been demonstrated in a neuroimaging study (Yovel et al., 2008) that reported a positive correlation between the size of the LVF bias and right hemisphere activation in areas specialized for face processing. However, more research is needed to replicate these findings.

Eye‐tracking methods are increasingly being used to measure the extent of LVF biases. Butler et al. (2005) used a gender recognition task to show that typically developing children made a greater number of fixations to stimuli in the LVF compared to the right visual field (RVF). Comparing looking time to stimuli has also been consistent with the LVF bias, with longer looking times to LVFs compared to RVFs reported when processing human faces (Butler & Harvey, 2006; Dundas et al., 2012; Guo et al., 2009). Increased looking time to the LVF has been associated with greater right hemisphere activation (Yovel et al., 2008), and the right hemisphere remaining dominant for face processing even when the face is no longer in the left visual field. One explanation for this effect is proposed by Dundas et al. (2012), who suggests that a tendency to attend more to faces in the LVF arises throughout development, and this facial information is predominantly processed in the right hemisphere. This right hemisphere specialization would lead to increased interest in facial information on the left, which results in a bias to focus on the left side of a person's face. Others have proposed a Right Hemisphere Theory (e.g., Borod, 1992), which suggests that the right hemisphere is dominant for social‐emotional processing more generally, regardless of modality. The original right hemisphere theory is well supported by a host of recent literature that is not modality specific and has identified a left bias for a variety of social touch phenomena (Ocklenburg et al., 2018; Packheiser et al., 2020) and cradling behaviors (Forrester et al., 2020).

A small number of eye‐tracking studies have examined when this preference and subsequent specialization emerges in typically developing children. Research suggests that a LVF bias develops sometime within the first 12 months (Guo et al., 2009; Wheeler, 2010), however, neither study employed a non‐face control in their stimuli, and it remains unclear whether these effects are face specific.

## Atypical lateralization

While atypical lateralization is not always a marker of underlying pathology, weaknesses, or even reversals in patterns are increasingly associated with divergences from typical development (Forrester & Todd, 2018), with support from fMRI research demonstrating direct neural correlates to support this behavioral bias narrative (Floris & Howells, 2018). LVF differences are reported in eye‐tracking studies with autistic children, who frequently show differences in face processing (Sasson, 2006) and social attention (Nelson et al., 2006). A lack of visual field bias, defined by a lower number of looks and shorter looking time to the left side of faces, was demonstrated at 6 months in children who went on to receive an autism diagnosis (Dundas et al., 2012). Other research suggests that a lack of LVF bias could extend to other forms of social attention and processing. Donati et al. (2020) compared the speed of looking and the number of looks to faces versus objects on the left or right side of a screen at 6 and 14 months. Autistic children showed a preference for stimuli on the right side and were slower to look to faces on the left. Associations were also found between gaze behaviors at 6 months and language and motor abilities at 14 months.

As mentioned above, children born early are more likely to develop atypical hemispheric lateralization, and there is a relation between lateralization differences, behaviors and cognitive abilities. Taken together, it is highly important to consider whether lateralization differences relating to atypical processing of social stimuli in preterm children early in development could contribute to differences in aspects of social development. One way to examine this is through comparing LVF biases. To our knowledge, no studies have focused on the development of a LVF bias as a marker of atypical hemispheric lateralization in preterm children. However, a handful of studies have identified other behavioral differences relating to lateralization. A large‐scale analysis of UK Biobank data found an association between handedness and birth weight, with lower birth weight associated with stronger atypical asymmetries (de Kovel et al., 2019). A meta‐analysis (Domellöf et al., 2011) reports a two‐fold increase in the likelihood of non‐right handedness in preterm children, as well as a relation between a lack of handedness and lower functioning across a range of cognitive domains. These preliminary findings suggest that behavioral markers may yield informative results about early divergences from typical development. It would be of particular relevance to understand these differences at the earliest age possible, to potentially influence the developmental trajectories of cognitive outcomes (Wetherby et al., 2007).

The purpose of the present study is to explore whether children born preterm, in comparison to children born at term, exhibit differences in the time they spend looking to faces versus objects in the left or right visual field. We analyzed data from two free‐viewing tasks featuring social and non‐social content, to assess whether term or preterm children exhibit a LVF bias to faces versus objects by 9 months of age. Based on the findings regarding preterm lateralization differences, we hypothesized that preterm infants will look less at faces on the left in terms of looking time and the number of looks.

## METHOD

### Nature of findings

A link between visual biases and social stimuli is well established in experimental psychology (e.g., Borod et al., 1997), but little is known about its developmental trajectory. Given that the current investigation demonstrates visual bias differences to social stimuli between preterm and full‐term infants, we add support to a growing body of empirical research hypothesizing that early motor sensory biases are both integral and critical to healthy cognitive development (for a review see Forrester & Todd, 2018). We would therefore recommend confirmation through future replication efforts.

### Participants

Participants for this study were recruited from the Theirworld Edinburgh Birth Cohort, a longitudinal study of outcomes after preterm birth (Boardman et al., 2020). Preterm infants (gestational age at birth <33^+0^ weeks) were recruited from the Simpson Centre for Reproductive Health (SCRH) at the Royal Infirmary of Edinburgh, by identifying women who presented with threatened preterm delivery at gestational age at birth <33 weeks. Healthy term controls were recruited from the SCRH by identifying women attending the SCRH to deliver at >37 weeks. In order to accurately assess developmental abilities, age corrected for prematurity was used for preterm infants. All infants were assessed across educational, cognitive and social domains. Families were invited to participate in eye‐tracking tasks and cognitive assessments (parental reports and standardized cognitive assessments) in infancy between 8 and 10 months. Participants were excluded from the study at the time of recruitment due to any of the following: major congenital malformations, chromosomal abnormalities, congenital infection, and infants with major overt parenchymal lesions (cystic periventricular leukomalacia, hemorrhagic parenchymal infarction) and posthemorrhagic ventricular dilatation. Ethical approval was obtained from the National Research Ethics Service (South East Scotland Research Ethics Committee) (NRES number 16 SS 0154). Written informed consent was confirmed by parents at the visit. All eye‐tracking and cognitive assessments took place at the Division of Psychiatry, University of Edinburgh.

Fifty‐one infants born preterm and 61 infants born at term completed the eye‐tracking tasks, at an average age of 9.1 months for preterm (corrected age) and 8.9 months for term infants. Table 1 provides participant demographics overall. Of the preterm group, 46 had been exposed to antenatal steroid for threatened preterm labor and 37 had been exposed to antenatal magnesium sulfate for neuroprotection. A total of eight infants had bronchopulmonary dysplasia (defined as need for supplemental oxygen at 36 weeks corrected gestational age), but none was oxygen dependent at the time of assessment.

**TABLE 1:** Participant demographics

Characteristic	Preterm (*n* = 51)	Term (*n* = 61)	*p*‐Value
Mean (range) gestational age at birth (weeks and days)	29^+3^ (24^+0^–32^+0^)	39 ^+6^ (36 ^+4^–42 ^+0^)	**<.001**
Mean (SD) birthweight (kg)	1.31 (0.37)	3.51 (0.48)	**<.001**
Median (IQR)* age (months)	9.01 (7.86–10.00)	8.94 (8.09–11.15)	0.519
Sex (M:F)	33:19:00	31:29:00	0.116
	1: 11.1%	1: 3.8%	
% SIMD quintiles	2–4: 63.9%	2–4: 56.6%	
	5: 25%	5: 39.6%	
Ethnicity			
Any White background	92.15%	90.20%	
White and Asian background	1.96%	1.63%	
White and Black African background	1.96%	0%	
White and Black Caribbean background	0%	1.63%	
Other Asian background	1.96%	1.63%	
Other mixed background	1.96%	4.91%	

IQR, interquartile range; SD, standard deviation; SIMD, Scottish Index of Multiple Deprivation 2015. Superscript numbers in the mean gestational age row represent age in days. As the data were not normally distributed, Mann–Whitney‐*U* was used to compare age at testing, mean birth weight, and gestational age at birth and SIMD, and Chi‐square was used to compare sex differences between groups. Bold values indicate significant *p* values.

### Eye‐tracking procedure

Two tasks were presented to participants. Both have been validated and described in detail in other reports (Gillespie‐Smith et al., 2016; Gliga et al., 2009; Telford et al., 2016). Participants were presented with two free‐viewing social paradigms, where trials were passively viewed and gaze patterns recorded. A fixed script ran identically each time, and each child saw the same trial order and the same stimulus order. Attention grabbers (in the form of colorful pictures on black backgrounds with sound effects) were presented in between blocks to maintain attention to the screen. Attention grabbers were gaze contingent to ensure attention to the onset of the new trial block.

### Eye‐tracking tasks

#### Pop‐out task

**FIGURE 1:**
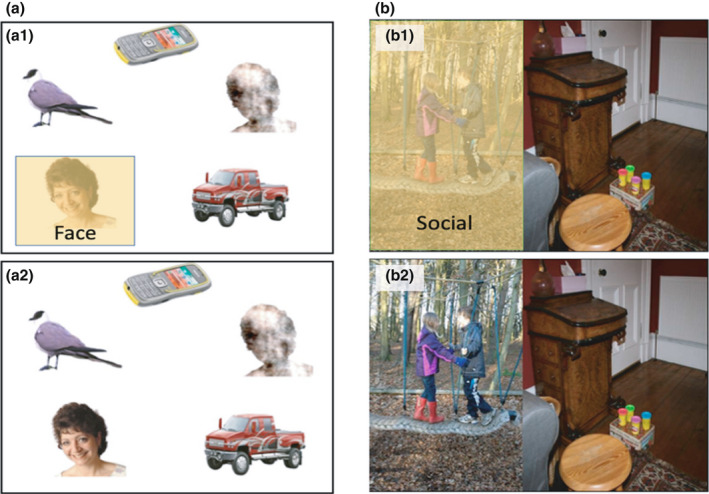
(a and b) The top row shows examples of one area of interest from the (1A1) face pop‐out and (1B1) social preference task. The bottom row (1A2 and 1B2) provides one example trial from each task

Each trial presented five images in a circular formation on a white background; two on the left, two on the right, and one in a central position, either above or below the central fixation point. A trial always featured one picture of a different human face, and four non‐social stimuli including a “face‐noise image” (consisting of an artificial scramble of a human silhouette, but not recognizable as a face), a car, a mobile phone and a bird (Elsabbagh et al., 2009). Each stimulus image measured 25 × 20 cm and there was a total of eight trials that were counterbalanced for vertical and horizontal locations of the face within the slide. Trials were viewed for 10 s each, and each block consisted of two or three trials, ensuring that no block contained a single trial. See Figure 1a for an example of an area of interest from a single trial in the pop‐out task.

#### Social preferential looking task (social preference)

Each trial consisted of a pair of photographs displayed side by side on the screen, both of which were real world scenes. One photograph in each pair showed a social scene (with people) and the other showed a non‐social scene (containing no people) (see Fletcher‐Watson et al., 2008). The photos were shuffled so that each social scene was paired with a different non‐social scene. Each trial was presented for 5 s, and each block contained four trials. Participants were brought back to the center between trials using colorful attention grabbers prior to the onset of a new pair of stimuli. A total of 12 trials were presented. Figure 1b shows an example of an area of interest from a single trial in the social preference task.

### Measures

Four observation variables were created for each task to compare looking times to social and non‐social stimuli on the left and right, as in Donati et al. (2020).

Face pop‐out: In order to focus on stimuli only from the left and right, stimuli presented in the center of the screen were removed from the analysis, leaving two different stimuli on the left and two on the right on each trial. Out of the eight trials, three trials contained faces on the left and two trials contained faces on the right. Three trials contained no faces on the left or right as they were positioned in the middle of the scene, and therefore removed from any left versus right analyses. All other stimuli were defined as non‐face stimuli. Therefore, four areas of interest (AOIs) were created: face left, face right, non‐face left, non‐face right.

Social preference: For each trial, two AOIs were created: social and non‐social. Stimuli were categorized as one of four observations: social left, social right, non‐social left, non‐social right.

For both the face pop‐out and social preference task, analysis was split by three measures, to understand the effects of side (left, right), object type (face or non‐face for the face pop‐out; social or non‐social for the social preference task) and clinical group (preterm, term).

### Observational variables

#### Looking time

Measured the total looking time to each AOI. The duration of all fixations made to a given AOI were summed and averaged across trials featuring the AOI.

#### Proportional looking score

In addition to looking time, a proportional looking time score was calculated (Telford et al., 2016). The proportional looking score was defined as the ratio of looking time to the area of interest (AOI) against looking time for the whole scene (proportional looking time = LT AOI/[LT to whole scene]). For all measures, trials were averaged across each participant.

#### Number of observations

The number of first looks to face left, face right, non‐face left and non‐face right were also measured. The attention grabber shown before the onset of each trial was not gaze contingent (i.e., the eye movements of the participant do not modify how long the attention grabber is on the screen or where the participant is looking). Therefore, first looks within the first 100ms were excluded as it is possible that a saccade was started before the image appeared (Liversedge & Findlay, 2000). Due to the small number of trials for face left and face right, it was not possible to calculate a proportion of trials on which participants looked at each AOI first. Instead, group frequencies were collected, and a proportion was created by dividing the number of first looks summed across participants by the number of valid trials for that same group of participants.

### Data cleaning and exclusion criteria

Due to poor data acquisition, 11 participants were removed from the face pop‐out dataset (seven preterm and four term), and five participants from the social preference task (three term, two preterm). Following the protocol of Telford et al. (2016), scores were calculated if a third of trials were valid. Trials with looking times <500 ms were excluded as they were not considered a sufficient quantity of data to represent the results of multiple eye movements to AOIs within a single trial (Gillespie‐Smith et al., 2016; Liversedge & Findlay, 2000; Telford et al., 2016).

### Analyses

SPSS 25 was used for all statistical analyses. AOIs were predefined according to previous protocols. Raw eye movement data were filtered into fixations using Tobii I‐VT classification algorithm (Dean et al., 2020).

Three‐way mixed ANOVAs were performed on looking time and proportional looking scores for the face pop‐out and social preference tasks to understand the effects of side (left, right), object type (face, non‐face for the face pop‐out; social and non‐social for the social preference task) and clinical group (preterm, term) on looking time. Homogeneity of variances was assessed by Levene's test for equality of variances. Normality was assessed using Shapiro–Wilks test of normality (*p *> .05), and visual inspection of QQ plots. Normally distributed data were analyzed using a three‐way repeated measures ANOVA. Data violating normality assumptions were log transformed. If transformed data did not pass normality assumptions, nonparametric, Mann–Whitney *U* tests were conducted. A Chi‐square test of independence was performed on the number of observations across clinical groups to identify any significant group differences in preferential looking patterns. All cell frequencies were greater than five.

### Association between looking time and first look

Due to the small number of trials for face left (three trials) and face right (two trials), it would not have been possible to reliably use first look data to calculate proportions of trials to each of the four observations per participant. However, previous studies have used looking time as a measure of biases (see introduction). Before proceeding with the analysis, we compared first look data to looking time data to demonstrate a relation between measurement types in two ways. (1) A Chi Square of independence was used to assess whether there was an association between the viewing activity to each observation (face left, face right, non‐face left, non‐face right) and measurement type across the trials. (2) A congruency measure was created to compare the location of first looks with the location that was viewed for the longest, per trial, per participant. For each trial, the location of the first look was compared with the looking time location. Trials were congruent if first look and looking time locations were the same, and incongruent if locations differed. We only included participants who had four or five trials for robustness to the evaluation (*n* = 62; preterm *n *= 28, term *n* = 34).

## RESULTS

### Comparison of data types

1. A Chi‐square of independence between the viewing activity to each observation (face left, face right, non‐face left, non‐face right) and measurement type across the trials was non‐significant, *X* ^2^ (1, *N *= 99) = .172, *p *= .678, suggesting no significant differences between measurement types in terms of viewing behavior. Table 2 provides mean group statistics to each AOI in the face pop‐out and social preference tasks.
2. A binomial sign test revealed significantly more congruent trials than incongruent trials between the looking time and first look locations (−6.433, incongruent—congruent, *p* < .001). Only two of 62 participants had more than 50% incongruent trials overall. We therefore found sufficient support for commonality in gaze patterns to looking time and first look measurements to proceed with subsequent analyses using looking time data as a measure of laterality.

**TABLE 2:** Descriptive statistics of the measure created for the face pop‐out and social preference tasks

Face pop‐out task	Preterm (*n* = 44)	Term (*n* = 55)
	Mean (SD)	Mean (SD)
Looking time (ms)		
Face left	2012.69 (1201.31)	2652.29 (1155.68)
Face right	2917.29 (1919.20)	2525.20 (1842.89)
Non‐face left	484.19 (271.41)	547.47 (300.47)
Non‐face right	467.71 (310.87)	509.33 (332.44)
Whole scene	3217.59 (1256.48)	3546.12. (1024.71)
Proportional looking time		
Face left	0.37 (.01)	0.47 (.15)
Face right	0.47 (.02)	0.43 (.21)
Non‐face left	0.08 (.05)	0.08 (.06)
Non‐face right	0.10 (.05)	0.09 (.05)
Social preference task	Preterm (*n* = 48)	Term (*n* = 58)
Looking time (ms)	Mean (SD)	Mean (SD)
Social left	1594.67 (656.89)	1950.88 (616.09)
Social right	1827.26 (726.95)	2046.48 (705.03)
Non‐social left	837.39 (439.44)	987.99 (481.11)
Non‐social right	937.13 (522.26)	935.52 (405.01)
Whole scene	3004.62 (647.45)	3061.45 (630.10)
Proportional looking time		
Social left	0.54 (0.18)	0.63 (0.12)
Social right	0.61 (0.20)	0.64 (0.15)
Non‐social left	0.33 (0.17)	0.31 (0.13)
Non‐social right	0.28 (0.16)	0.21 (0.15)

### Looking time

#### Face pop‐out

**FIGURE 2:**
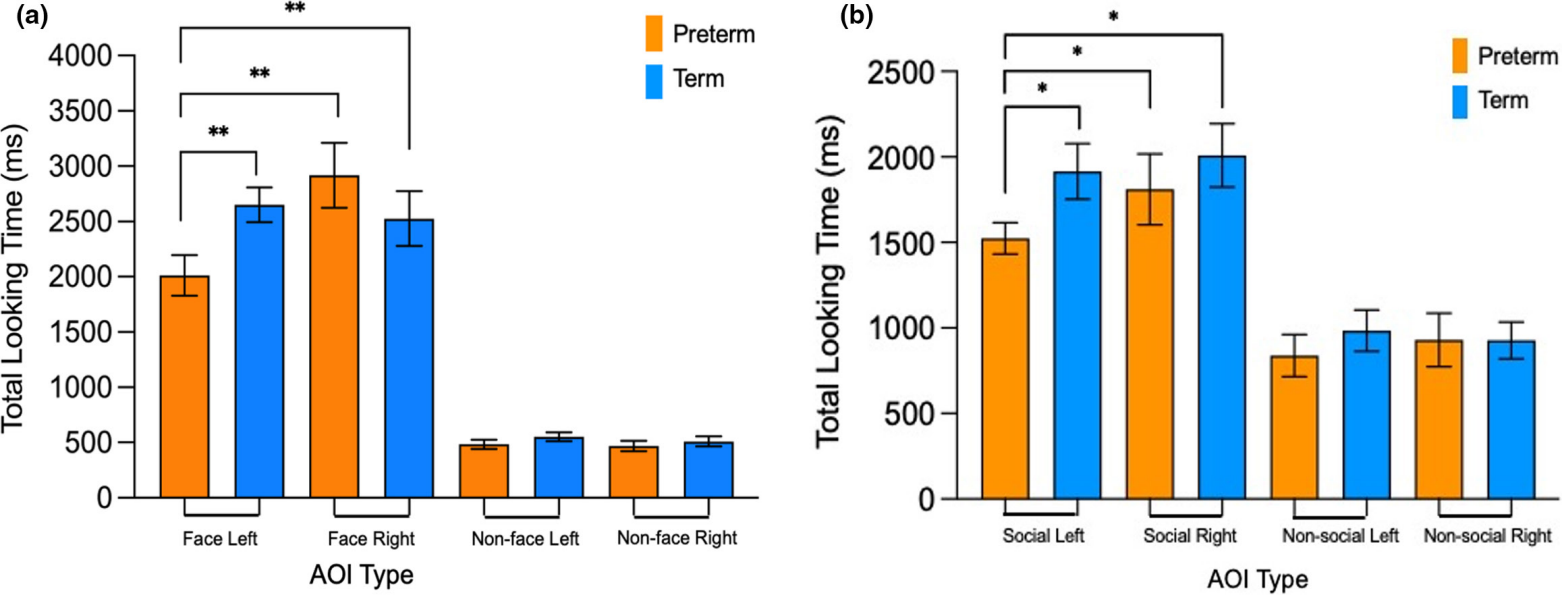
(a) Looking time to the four observations in the face pop‐out task. (b) Looking Time to the four observations for the social preference task. Error bars represent the standard error of the mean. * indicates significant post hoc comparison at *p* < .05, ** indicates significance at *p* < .01

For the whole sample, there was a significant three‐way interaction between side, object, and clinical group, (*F*(1, 96) = 7.817, *p *= .006, partial *η* ^2^ = .064). There was also a significant main effect of side *F*(1, 96) = 14.435, *p *≤ .001. For term infants, there was no significant two‐way interaction, *F*(1, 54) = .133, *p *= .717. For preterm infants, there was a significant two‐way interaction between side and object *F*(1, 42) = 12.218, *p *= .001. Preterm infants spent significantly less time looking to face stimuli on the left compared to the right (*p* < .001; *d* = 0.58, 95% CI, 349–1137) with a mean difference between looking times of 904.6 ms (2012.69 vs. 2917.29). Figure 2a shows mean looking times to the four AOIs in the face pop‐out task, with significant post hoc differences indicated.

### Social preference

For the whole sample, there was no significant three‐way interaction between slide, clinical group and object (*p* = .141). There was a significant two‐way interaction between side and clinical group *F*(1, 106) = 3.995, *p *= .048 partial *η* ^2^ = .039, and a significant main effect of object (*F*(1, 104) = 276.63 *p *≤ .001, partial *η* ^2^ = .727). The preterm group spent significantly less time looking at social objects on the left than on the right (*p* = .047, *d *= 0.49, 95% CI, 3.78–486), with a mean difference in looking times of 232.59 ms (1827.26–1594.67), and less time looking to social objects on the left compared to term infants (*p* = .002, *d *= 0.56, 95% CI, 165.72–658.88), with a mean difference between looking times of 356.21 ms (1594.67 vs. 1950.88). There was no group difference in looking time for social objects on the right (*p *= .187). Figure 2b shows the mean looking times to the two AOIs in the social preference task, with significant tests indicated.

### Proportional looking time

#### Face pop‐out

For all participants, a significant three‐way interaction between side, object and clinical group was found *F*(1, 101) = 7612, *p *≤ .001, partial *η* ^2^ = .070 and a main effect of object *F*(1, 45) = 75.504, *p *≤ .001. For the preterm group, there was a significant two‐way interaction between side and participant type *F*(1, 45) = 7.293, *p* < .017. Preterm infants spent proportionally less time looking to face stimuli on the left versus face stimuli on the right. (*p* ≤ .025, *d *= 0.47, 95% CI, .011–.17), with a mean difference in proportional looking times of 6.40 (.37–.47).

#### Social preference

Mann–Whitney U tests were used to analyze social preference proportional scores, as raw and transformed data violated normality tests. Proportional looking time scores to social left were significantly different between preterm (Mdn = .578) and term infants (Mdn = .650), *U* = 1043, *Z *= 2.336, *p *= .019. Proportional looking time scores were not significantly different between groups for social right, non‐social left, or non‐social right. see Table 2.

### Number of observations

**FIGURE 3:**
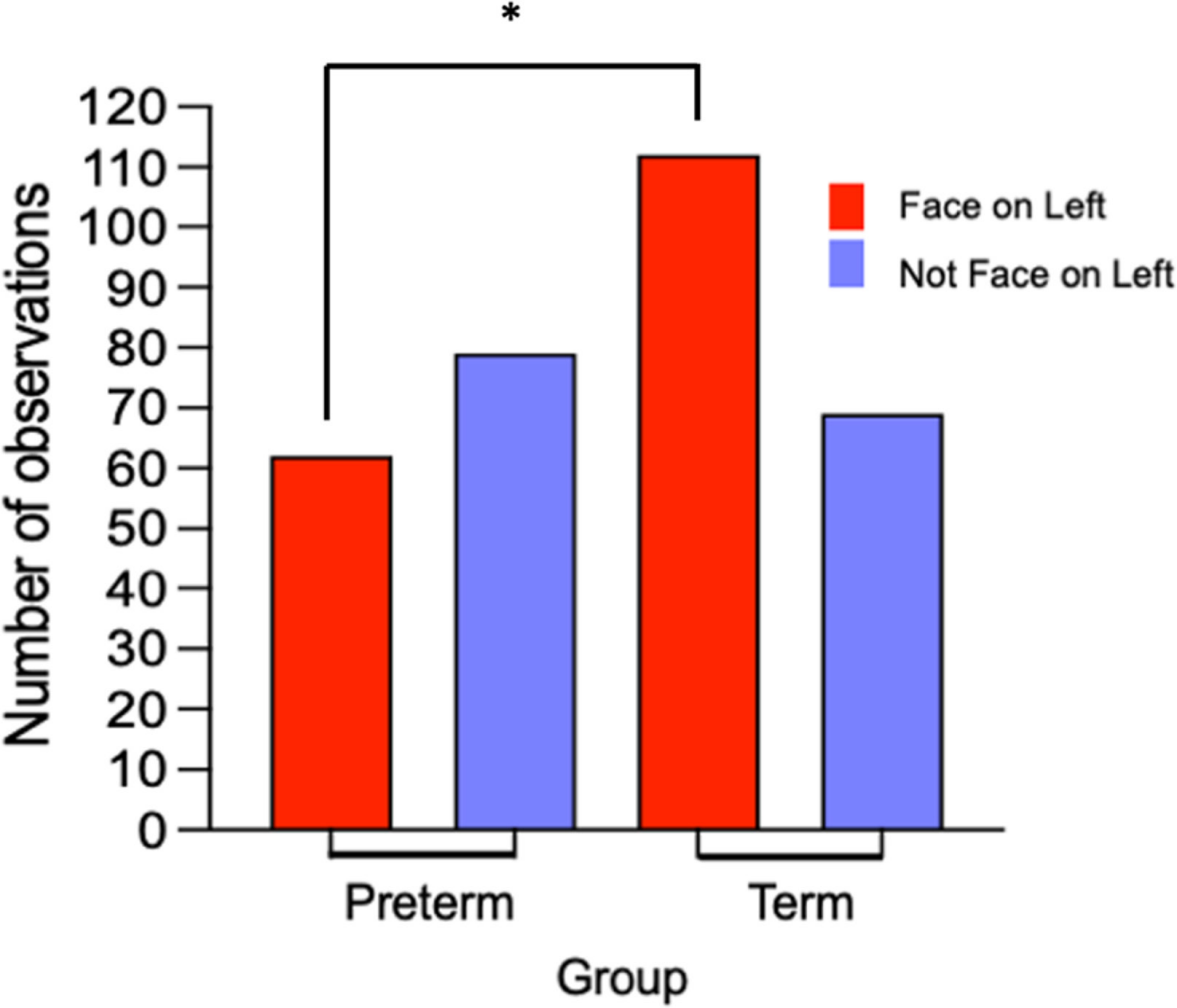
Group frequencies of first look to face left versus other AOIs. * indicates significant comparison at *p* < .05

In the face pop‐out, there was a significant association between number of first looks to face left and group (*χ* ^2^(1) = 7.618, *p *= .006). Figure 3 shows that preterm infants were making significantly fewer first looks to face left versus other stimuli compared to term infants. For the social preference task, there was no significant difference in the number of first looks to face left between preterm and term infants.

## DISCUSSION

This study demonstrates for the first time, that infants born preterm show a distinct gaze profile for processing faces and social stimuli in the left versus right visual field, compared to term‐born infants. These differences are indicative of atypicalities in the development of visual field biases, shown by a reduced preference for viewing social stimuli in the LVF. This finding was broadly consistent across two previously validated free‐viewing social tasks, and across three different measures of viewing preferences (Dean et al., 2020; Telford et al., 2016).

Across looking time and proportional looking time scores, we reported a main effect of object and a two‐way interaction between side and clinical group in both tasks, and a three‐way interaction between side, object and group in the face pop‐out task. In terms of the main effect of object across paradigms, both term and preterm infants exhibited a significant preference for looking more toward face or social stimuli, compared to non‐face or non‐social stimuli. This supports previous findings that infants demonstrate preferential looking toward faces or social stimuli over non‐social objects (Gliga et al., 2009; Telford et al., 2016). Crucially, this finding was specific to face and social stimuli, which suggests that differences in looking time cannot simply be attributed to attentional processes.

Children born at term showed no significant differences in looking time or the number of looks to faces in the left versus right visual field, across either task. This suggests that as a group, the term infants had not developed a LVF bias by the 9‐month timepoint (though children were aged between 7 and 10 months here). This finding is supported by previous eye‐tracking studies in typically developing infants suggesting that the LVF bias generally develops between 6 and 12 months (Wheeler, 2010), with the majority of research finding the LVF in children older than 9 months, but within the first year of life (Dundas et al., 2012; Guo et al., 2009). The current study therefore provides a first contribution toward understanding the development of the LVF bias in infants born preterm without a known neurodevelopmental disorder.

Preterm infants spent significantly less time looking at social or face stimuli on the left compared to the right, and the two and three‐way interactions were primarily driven by these viewing patterns. We found the same effect when comparing the number of looks to face stimuli on the left versus right in the face pop‐out task in preterm infants, but no significant difference in the social preference task. However, the AOIs for the social preference task were more general than the face pop‐out, with each AOI (social, non‐social) covering half of the screen. This lack of specificity could be one reason why the number of looks were not significantly different here.

There was no difference between infant and preterm looking times or proportional looking scores to faces or social stimuli on the right, whereas looking time to social scenes or faces on the left was significantly diminished in preterm infants. This reduction in left looking is the opposite pattern that we would expect to see in the typical development of visual attention. Research has established that information from social stimuli presented in the LVF is projected to the right hemisphere for visuo‐spatial processing which contributes to the specialization of socioemotional processing in the right hemisphere (Vallortigara & Versace, 2017). It is possible that later in development, preterm infants will exhibit a trajectory that is comparable to children born at term that would be suggestive of a developmental delay. If this were the case, we would expect to see children born at term to show this same pattern of non‐left looking earlier in development. However, given that research has identified a reduction in handedness that is hypothesized to be a behavioral marker for some aspects of altered hemispheric lateralization (Domellöf et al., 2011) and physiological data linking atypical lateralization and a lack of LVF (Floris & Howells, 2018), it is likely that the LVF bias will not develop typically in preterm infants. It is therefore possible that this finding with preterm infants could be representative of a physiological marker of early processing differences, and indicative of emerging lateralization differences that are seen across the lifespan in people born preterm (Lee et al., 2021).

However, it is currently unclear whether this is a manifestation of biology or environment that influences the early processing differences indicative of emerging atypical lateralization. For example, Vervloed et al. (2011) showed that adults who were cradled on the right by their mothers in infancy developed a weakened LVF bias for identifying faces and facial expressions—although findings were within the typical variability of the population. This suggests that environmental factors, such as early parent‐infant interactions could interfere with typical development of lateralized brain functions (Malatesta, Marzoli, Apicella, et al., 2020; Malatesta et al., 2020). In relation to the current findings, research suggests that the left‐cradling bias can also be disrupted in mothers who are separated from their children after delivery (Salk, 1970). As parents of preterm children are likely to be separated from their child for a time after birth (Treherne et al., 2017), this could be one factor that contributes to the lack of left looking shown in the preterm group.

A lack of left looking or reversal in orienting behaviors across eye‐tracking paradigms has also been demonstrated in autistic children (Dundas et al., 2012) and infants with an elevated familial likelihood of being autistic, who go on to receive a diagnosis later in development (Donati et al., 2020). Furthermore, a reduced interest toward social stimuli in the current study could relate to differences found in attentional patterns to social stimuli in newborn infants with an increased familial likelihood of autism (Di Giorgio et al., 2016).

The prevalence of autism in preterm infants is estimated to be around 7 percent (Agrawal et al., 2018), so while slightly elevated from the general population where the prevalence is estimated to be around 1% (Elsabbagh et al., 2012) it is unlikely that this finding represents a subgroup of preterm children who will go on to receive an autism diagnosis. There was no evidence for subgroups or extreme values within the preterm sample. However, we could interpret these laterality differences as an indication of a general developmental delay across autistic and preterm populations; where a foundational and critical element of typical brain development that when disrupted, can be associated with a myriad of neurodevelopmental conditions (although the causal direction is not yet known).

This is the first study to look at the development of a LVF bias in preterm infants. In comparison to infants born at term, preterm gaze patterns showed a reduced interest in faces and social stimuli on the left across tasks, which suggests the possibility of early differential specialization. It is important that future research investigates whether this finding is replicated in other samples, whether differentiation continues later in development, and the potential link to other aspects of cognition. Recruitment took place in one hospital in Edinburgh, UK, which is less ethnically diverse than other large cities, so future replications recruiting from ethnically diverse populations will be beneficial to strengthen generalizability of these findings.

It would be important to understand whether reduced looking to the left is indicative of generalized developmental delay, or whether autistic children, and children born preterm, exhibit different developmental trajectories and potential compensatory mechanisms. Comparing the neural correlates of visual field biases in these children could be particularly beneficial to identify neurological similarities or differences between these groups of children and the associations with gaze behaviors.

It would also be important to consider additional factors that could alter hemispheric lateralization that could relate to preterm birth. For example, early life stress has been identified as a commonality between changes in asymmetries and neurodevelopmental conditions (Berretz et al., 2020) and as an influence on brain structures that underlie socioemotional development (Stoye et al., 2020). Therefore, future studies could evaluate early life stress in developmental outcomes.

Understanding the associations between behavioral and brain asymmetries, and potential links to other aspects of development could offer unique breakthroughs in therapeutic practice and interventions. Identifying divergences at the earliest time points could yield the greatest developmental gains for children born preterm, and our innovative findings here could reflect a first step in identifying early behavioral divergences.

## Data Availability

The original data supporting this study are accessible under the terms of the Data Access and Collaboration Policy for the Theirworld Edinburgh Birth Cohort. For details, see: http://www.tebc.ed.ac.uk/2019/12/data‐access‐and‐collaboration/
